# Current News

**Published:** 1901-06

**Authors:** 


### Current News.

### PENNSYLVANIA STATE DENTAL SOCIETY.

The Council of the Pennsylvania State Dental Society have deemed it advisable to change the next meeting-place from Pittsburg to Ligonier, Pa. The Committee is arranging for proper accommodations, railroad rates, etc., and anticipate one of the very largest and best meetings in the history of the Society. Programmes will be issued not later than June 15.

V. S. Jones,
*Corresponding Secretary.*

---

### MASSACHUSETTS DENTAL SOCIETY.

The thirty-seventh annual meeting of the Massachusetts Dental Society will be held in Massachusetts Institute of Technology Building (Huntington Hall), Boston, Wednesday and Thursday,

June 5 and 6, 1901. The committee have secured a goodly amount of talent, and the meeting promises to be well worth the consideration of the profession.

EDGAR O. KINSMAN,

*Secretary.*

CAMBRIDGE, MASS.

---

### NEW JERSEY STATE DENTAL SOCIETY.

THE thirty-first annual session of the New Jersey State Dental Society will be held in the Auditorium, Asbury Park, N. J., commencing Wednesday, July 17, 1901, at ten A.M., and continuing in session Thursday and Friday. The Columbia, adjoining, will be the head-quarters, with rates of $2.50 and $3.00 per day.

To the busy practitioner who desires to witness the latest and best efforts in clinical dentistry, "Come." Fifty clinics. The best and newest efforts in the science of dentistry. Come and hear five papers read. A veritable museum of the latest and best in electrical appliances, mechanical tools, the chairs, instruments, and accessories of the modern dental office. Come and see us, and mark the days off *now.* The time will not be wasted. You will see the contents of not only one dental depot, but of all the country. The best efforts of the inventions pertaining to our profession up to date. The city dentist as well as the one from the cross-roads will see and learn something.

CHARLES A. MEEKER, D.D.S.,
*Secretary.*

29 FULTON STREET, NEWARK, N. J.

---

### TENNESSEE DENTAL ASSOCIATION.

THE next meeting of the Tennessee Dental Association will be held jointly with the Southern Branch of the National Dental Association, at Nashville, Tenn., beginning Monday July 29, 1901. The profession is cordially invited to be present.

W. M. SLOCH, Memphis, Tenn.,
*President.*

A. SIDNEY PAGE, Columbia, Tenn.,
*Secretary.*

### COLORADO STATE BOARD OF DENTAL EXAMINERS.

The Board of Dental Examiners for the State of Colorado will meet in Denver, June 3, 1901, at ten A.M., to examine applicants for license. In addition to the usual written and oral examination, candidates must supply their own patients, and come prepared with all necessary instruments, rubber dam, and gold, to perform practical operations under the supervision of the board, which will pass on suitable selections of cavities.

H. F. Hoffman,
*Secretary.*

611 California Building, Denver, Col.

---

### NATIONAL ASSOCIATION OF DENTAL EXAMINERS.

The nineteenth annual session of the National Association of Dental Examiners will be held at the Plankington Hotel, Milwaukee, Wis., Friday, August 2, 1901, commencing at ten A.M. and continuing Saturday and Monday. Arrangements are under way for those residing in the Middle and Eastern States for a reduced fare by the Lehigh Valley Railroad from New York and Philadelphia. Full particulars in the later journals.

Charles A. Meeker, D.D.S.,
*Secretary.*

29 Fulton Street, Newark, N. J.

---

### NORTH CAROLINA STATE BOARD OF DENTAL EXAMINERS.

The North Carolina State Board of Dental Examiners will meet in Morehead City, N. C., June 24 and 25, 1901. The examination will be written, together with practical operations in the mouth. Applicants are required to furnish materials and instruments.

R. H. Jones,
*Secretary.*

Winston, N. C.

### SOUTH DAKOTA STATE DENTAL SOCIETY.

The South Dakota State Dental Society will meet at Sioux Falls, June 11, 12, and 13.

B. S. Blunt.

---

### NEW ENGLAND ASSOCIATION OF DENTAL EXAMINERS.

At the Algonquin Club, Commonwealth Avenue, Boston, on Wednesday evening, April 24, this Association assembled for its fifth annual dinner and meeting. The discussion after the dinner was upon the conduct of State boards and their mutual relations, the object of the organization being the creation of a standard examination in all the New England States, so that there can be, under suitable restrictions, an interchange of certificates. All the State boards were well represented, and the honorary members, formerly members of State boards, present were Dr. L. D. Shepard, of Boston; Dr. J. Scare Hurlburt, of Springfield; Dr. C. A. Brackett, of Newport; and Dr. A. B. Miller, formerly of the Maine board. The dean of the Dental Department of Harvard, Dr. Eugene H. Smith, and Dr. Harold Williams, dean of the Dental Department of Tufts College, were the guests of the evening.

The following were chosen officers for the ensuing year: President, Dr. Thomas J. Barrett, of Worcester, Mass.; Vice-President, Dr. Dana W. Fellows, of Portland, Me.; Recorder, Dr. George L. Parmele, of Hartford, Conn.; Chairman of the Executive Committee, Dr. John F. Dowsley, of Boston, Mass.

